# Myoelectrically Controlled FES to Enhance Tenodesis Grip in People With Cervical Spinal Cord Lesion: A Usability Study

**DOI:** 10.3389/fnins.2020.00412

**Published:** 2020-05-05

**Authors:** Rune Thorsen, Davide Dalla Costa, Ettore Beghi, Maurizio Ferrarin

**Affiliations:** ^1^Biomedical Technology Department, IRCCS Fondazione Don Carlo Gnocchi, Milan, Italy; ^2^Neurorehabilitation Unit, ASST Grande Ospedale Metropolitano Niguarda, Milan, Italy; ^3^Department of Neuroscience, Istituto di Ricerche Farmacologiche Mario Negri IRCCS, Milan, Italy

**Keywords:** functional electrical stimulation, rehabilitation, tetraplegia, assistive technological devices, neuroprosthesis, activities of daily life

## Abstract

People with tetraplegia are often lacking grip strength, causing impairment in activities of daily living. For them, improving hand function is a priority because it is important for autonomy and participation in daily life. A tendon transfer surgery may be an option to improve the tenodesis grip, but it is an invasive procedure. Alternatively a similar effect can be produced, using a non-invasive method. We have previously described how myoelectrically controlled functional electrical stimulation (MeCFES) can be efficient for enhancing grip strength, using a one channel research prototype with wired connections to surface electrodes. In this paper we focus on the usability for activities of daily living and how it can fulfill an actual need. We recruited 27 participants with a cervical spinal cord lesion (C5-C7) for this trial. They tested the device in 12 sessions of 2 h each, in which the participants performed self selected activities involving the tenodesis grip. User centered outcomes were validated questionnaires: the Individually Prioritized Problem Assessment (IPPA) and the Quebec User Evaluation of Satisfaction with Assistive Technology (QUEST). Furthermore, they were asked if they found the device useful for continued use in daily life. The device facilitated prioritized activities for all participants. The IPPA change score was 4.6 on average (STD:3.5, effect size:1.3), meaning that the system greatly facilitated problematic tasks and the large effect size evinces that this was a meaningful improvement of hand function. It compares to the impact that a mobility device like a wheelchair has on daily living. Fourteen subjects found the system useful, expressing the need for such a neuroprosthesis. Examples of acquiring new abilities while using the device, indicate that the method could have a therapeutic use as well. Furthermore, results from the IPPA questionnaire are indicating what issues people with tetraplegia may hope to solve with a neuroprosthesis for the hand. The satisfaction of the device (QUEST) indicates that further effort in development should address wearability, eliminate wires, and improve the fitting procedure.

## Introduction

Cervical spinal cord lesion results in paralysis of most of the body, including the arms and legs, and is called tetraplegia. The clinical records should contain the last intact neurological level and the AIS classification (i.e. completeness) of lesion (American Spinal Injury Association, 1992). This is a key to understanding what residual functions may be spared by the lesion.

The prevailing level of lesions are C6, C5, and C7 which will, depending on the severity of lesion, impair the hand function to some extent (Winslow and Rozovsky, 2003; Snoek et al., 2004; Thorsen et al., 2014). Most will have proximal control but no or reduced finger flexion force, thus having difficulties in grasping objects (Yarkony et al., 1988; Mulcahey et al., 2007).

For that group, the ability to perform activities of daily living (ADL) is severely impaired and therefore regaining hand function has a high priority (Snoek et al., 2004; Anderson, 2004).

The wrist grip or tenodesis grip is a functional passive hand grasp that is often promoted by a conservative treatment early in rehabilitation: “The grip is obtained by first allowing gravity to flex the wrist when the fingers and thumb fall into extension. The hands or the first finger and thumb are placed over the object to be lifted. Extension of the wrist by extensor carpi radialis places passive tension on the flexors and enables a light object to be held in position. If the object is heavier, the pull of gravity can be partially overcome by supinating the forearm. The grip is achieved through the combined control of the unaffected muscles with the passive tension in the finger and thumb muscles” (Bromley, 2006).

However, this is often only providing a weak grip. Some may be candidates for surgical interventions where muscle tendons are transferred to assist the grip (Mulcahey et al., 2007). Such interventions apply only to well selected patients, are very invasive and require extensive resources. There is a risk of weakening the donor movement and the functional outcomes are not always predictable. Not all people are suitable for surgery and some are not willing to undergo such invasive intervention as they may feel insecure about the outcome (Bryden et al., 2012; Dunn et al., 2012).

Though volitional control of the finger flexors is absent, the muscles may still be innervated and can therefore be stimulated to elicit tetanic contraction. When the tenodesis grip is used, the wrist extensors are producing a myoelectric signal as a result of this voluntary muscle contraction (VMC). The VMC can be estimated from the myoelectric signal and used to control stimulation of the finger flexors like a sort of virtual tendon transfer. Both recording of myoelectrical signals and functional electrical stimulation are common in clinical use (Benton et al., 1980; Hermens et al., 2000). However, myoelectrically controlled functional electrical stimulation (MeCFES) requires specialized electronic equipment and processing and a major challenge is to estimate the users intent of volitional contraction, the VMC (Thorsen, 1997). The VMC is present as a low-level stochastic signal in the recorded signal. During stimulation the recorded signal will be mixed with a noise coming from the stimulation responses which are magnitudes stronger. Each stimulation pulse generates a muscle twitch as well as direct and indirect electrical responses. The principal direct responses are spill-over of the stimulation current to the amplifier input. This stimulation artifact can be reduced by electrode placements and by using perfectly biphasic stimulation (Thorsen and Ferrarin, 2009). Another component of the signal is the compound muscle unit action potential, an electrical response from the muscle to the stimulation of the nerve. Both are quasi-stationary signals (Thorsen, 1997). Indirect responses to stimulation are randomly occurring H-reflexes, F-waves and motion artifacts caused at the electrode interface (Thorsen et al., 2005). The amplifier circuits and the signal processing must attenuate stimulation responses to isolate the signal from which the VMC can be estimated (Thorsen, 1997). The instant stimulation level is calculated as the VMC estimate multiplicated by a gain factor. A too high gain may result in an unstable system because the stimulation response is generating a positive feedback path to the VMC estimate. This may result in saturation of the stimulation causing the system to latch up to a full output state (full-on). However, with careful adjustment of these parameters, the user may obtain a continuous control which allows her/him to control the resulting grip strength as previously demonstrated with tracking tests (Thorsen, 1997; Thorsen et al., 2001).

The MeCFES is different from similar approaches, such as EMG triggered FES (Sun et al., 2018) because the user has direct and continuous control of the resulting FES in terms of onset, duration and intensity. Therefore it has potentially other useful applications, as for example in rehabilitation of the hemiplegic hand in stroke patients (Jonsdottir et al., 2017).

We have shown that MeCFES assisted tenodesis grip can provide functional benefits and possibly also sustained improvement (therapeutic effect) of the hand function (Thorsen et al., 2013). That report focused on the functionality and efficiency in terms of performance, but the ultimate goal of an assistive device is to enable the user to participate in desired activities. Therefore we must also consider its usability in daily living (de Witte et al., 2018). For that purpose two validated instruments are frequently used: the Individually Prioritized Problem Assessment (IPPA) for assessing the perceived effectiveness (Wessels et al., 2002; Pettersson et al., 2006; Salatino et al., 2018), and the Quebec User Evaluation of Satisfaction with Assistive Technology (QUEST) which aims to capture the individual’s satisfaction (Demers et al., 2002; Salatino et al., 2018).

This paper serves to elucidate usability and need for MeCFES as an assistive device; identification of the users ADL priorities, perceived efficiency for resolving ADL problems, what it can be used for, who the users could be, detecting technical issues and where to focus further development of a useful neuroprosthetic device for increasing the autonomy in daily living for people with tetraplegia.

## Methods

Candidates for participation in this study were selected through screening of the database of clinical records at the two participating spinal units of the Lombardy region of Italy. Chronic patients with lesions at C5 – C7 AIS A-D were contacted, informed and invited to participate in the device testing [further details in Thorsen et al. (2013)]. The study was approved by the centers’ ethics committees, the Ministry of Health and all participants signed the approved consent form. After receiving full information about the system and its purpose, candidates decided together with the medical team if they were eligible and wanted to participate.

### Participants

Participation in the study was voluntary and in adjunct to whatever other treatments they received at the spinal units. Therefore the resulting participants were people who had time, energy and could easily come to the rehabilitation department of the spinal units.

The criteria for eligibility were: 1. A functional hand: an opposition of the index finger and the thumb; 2. A wrist posture that determines the position of the thumb and fingers allowing the finger to come in contact with the thumb; 3. Innervation of at least one of the extrinsic flexors (flexor digitorum superficialis, flexor digitorum profundus, flexor pollicis longus).

Each unit had two medical doctors performing recruitment and assessments supported by a technician. The medical doctors verified the neurological level, the functional status of the upper extremity and the MeCFES was used for testing if the muscles could be stimulated to produce a useful grip. Those subjects in which the stimulation resulted in a useful grip were enrolled for a trial period (twelve sessions of 2 h) if they were willing and able to participate in the study. During these sessions the device would be used actively as an assistive device to practice activities of daily living under the guidance of an occupational therapist who kept notes about activities, issues and settings.

### Material

**FIGURE 1:**
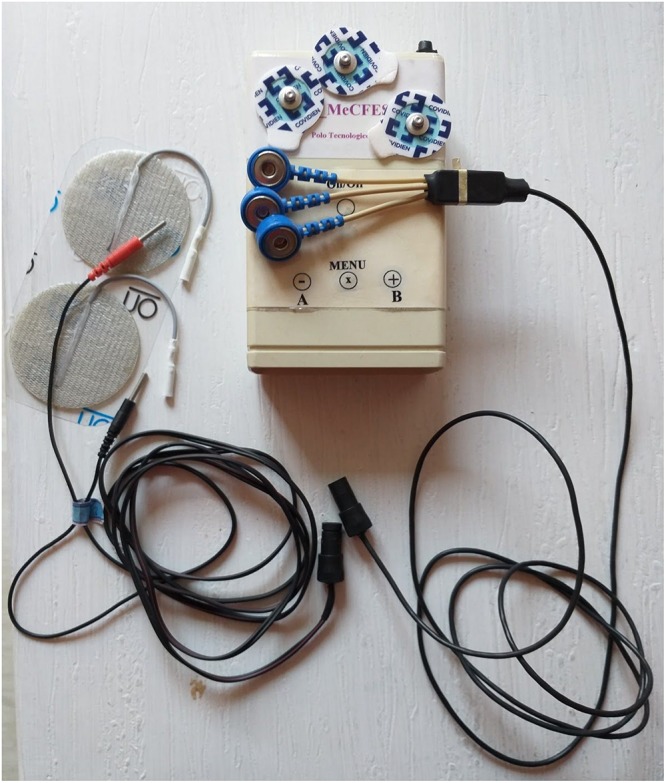
The device with cables and the adhesive electrodes.

A small series of pocket size battery powered custom made prototypes incorporating one channel MeCFES were built. They were equipped with two cables for connecting to a pair of recording electrodes (standard ECG electrodes) and a pair of stimulation electrodes (standard TENS electrodes) see Figure 1. The amplifier circuit was built around two instrumentation amplifiers (AD620) with a non-linear DC-compensation feedback, in a configuration that optimized the recovery from saturation and motion artifacts, see Thorsen, 1999 for details and design files of the system are published in the repository https://github.com/thorsenrune/mecfes. After a second order bandpass filter (16–500 Hz) the amplified (60dB) signal was converted by a 16bit ADC with oversampling at 2000 Hz, resulting in bins of 120 samples between consecutive stimuli (the inter stimulation interval). Two blanking intervals were applied. The first blanking interval was discarding the first 20 samples to reduce the stimulation responses. The second blanking interval was preset to discard 20 samples around the 80’th sample where F-waves or H-reflexes may typically be found. Then a first order comb filter with notches corresponding to the interval between stimulation pulses (60 ms) was applied to the signal to remove the quasi-stationary component of the remaining stimulation responses. Then the average rectified value was calculated over each 120 samples bin and fed to a first order IIR lowpass filter with a variable cutoff frequency (default to 1 s). This would be the estimated level of volitional muscle contraction (VMC). Finally a piecewise linear function (PWL) was applied to calculate the instant stimulation amplitude from the VMC. The PWL had an offset (i.e. the VMC from which stimulation would start), a gain (relation between wrist extension and stimulation) and a maximum stimulation level. The lowpass filter setting would be a tradeoff between response time and precision of the control.

These settings could be adjusted individually for each participant and were retained in the device memory for the next use, thus the device could operate as a self contained unit.

**FIGURE 2:**
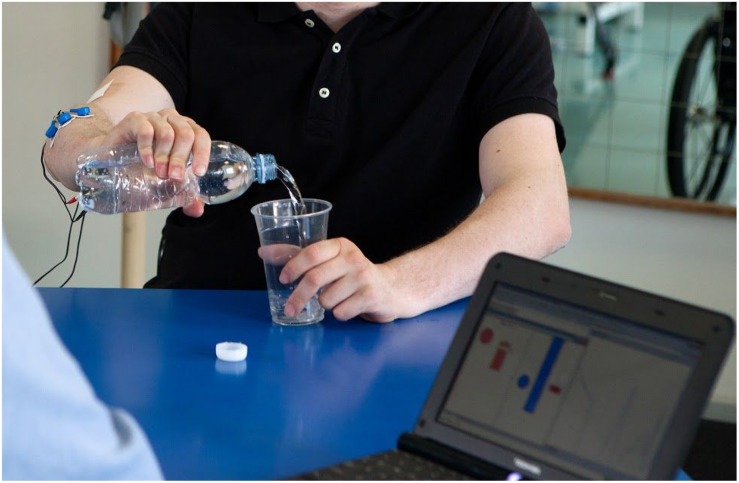
A participant using the MeCFES for pouring water into a glass. The laptop is used for monitoring the signals and settings of the device.

Two types of interfaces for adjusting these parameters were implemented. One method was through a graphical user interface (GUI) on a laptop, see Figure 2, which could be connected to the device via bluetooth. The other method was to use three tactile switches (buttons) on the device itself, besides the on/off switch, see Figure 1. The device also comprised a buzzer for acoustic feedback of current operating state. The three buttons were supposed to be simple to use, inspired by the classic digital wrist watch type menu (mode) and plus/minus adjustment buttons. They were low force (1.5N) tactile switches allowing for light touches to activate them. The main button was the mode button. A short press would switch between stand by and stimulation mode. A short press on the mode button would toggle between pause (no stimulation output) or active (MeCFES control of stimulation output), whereas a long press would cycle modes for adjusting the three key parameters (offset, gain and maximum stimulation) for each user. The pause mode served to quickly suspend stimulation and if the user was performing activities where the MeCFES was not needed e.g. resting or not performing activities which involved grasping.

The GUI would allow more granular control and could display the myoelectric signal in it’s raw and filtered form. Visual sliders were used to change the parameters and show instant stimulation (mA) and the VMC (uV). For advanced uses the blanking interval could be adjusted with indicators on the filtered signal.

### Electrode Placement & Setup

The fitting procedure for placing electrodes and setting up the device was as follows.

**FIGURE 3:**
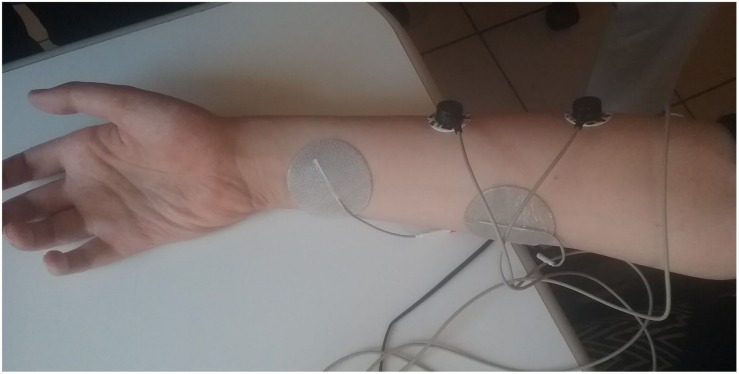
An example of the electrode position on one subject.

First the motor points for the extrinsic finger flexors, primarily the flexor digitorum superficialis (FDS) and the flexor pollicis longus (FPL) should be located, but also the flexor digitorum profundus (FDP) and eventually indirect activation of the intrinsics could be valid possibilities, as long as a functional grasp was obtained. One electrode was placed over the muscle belly of FDS, which is one third proximal on the anterior side of the forearm and the other over the distal part of the FPL (see Figure 3). Using the MeCFES the stimulation could be manually ramped up to establish both maximum stimulation and resulting activation of finger flexion. Electrodes could be moved by trial and error until a satisfactory position was found which allowed for a good firm grasp at a tolerable stimulation level (the maximum stimulation level).

Then recording electrodes should be placed over the wrist extensors. The wrist extensors are the extensor carpi radialis longus, brevis and the extensor carpi ulnaris, which are fusiform muscles with the muscle belly around one third on the proximal part of the dorsal side of the forearm. The actual muscle to use for EMG recording depends on the lesion. It can be located by palpating/touching the muscles during voluntary active wrist extension. The important issue here was that volitional wrist extension was going to control finger flexion so stimulation intensity would be depending directly on the degree of active wrist extension resulting in an augmented key- or palmar grip strength.

As it is important that the recorded signal is of good quality and free from noise including stimulation responses, the GUI could be used to inspect the recorded signal. After placing the electrodes and establishing the maximum stimulation the gain should be adjusted to allow for the proportional control. Finally the offset should be set so no stimulation was given when the user relaxes the wrist extensors. These procedures were described in the investigators brochure and explained to participating clinicians.

### Outcomes

We selected two validated outcome measures to quantify the efficiency in alleviating limitations on functioning with respect to the personal priorities (IPPA) and to identify issues that should be assessed for further development (QUEST).

Individually prioritized problem assessment consists of two interviews (Wessels et al., 2002). Initially the user is asked to identify up to seven problem items that he or she experiences in everyday life; activities that the device should make less difficult. In this interactive process the problems are identified in relation to concrete daily activities. In the first (baseline) interview the interviewee will rate the importance of each problem from 1 (not important) to 5 (very important) and it’s difficulty from 1 (easy) to 5 (impossible). The baseline score is the mean of importance times difficulty ranging from 1 to 25. After having used the assistive device for a period the same problem items are rated again with a new scoring, but this time rating the difficulty in performing the items when assisted by the device. This follow up score is again the mean of the importance times difficulty. However, since the importance score is the same as in the baseline interview it can be demonstrated that the change score (baseline-follow up) in theory may range from −20 to 20. An extreme negative score would imply that all tasks were very important but easy to perform and that the device made them all impossible. A positive maximum change score would imply that all important, but impossible tasks were rendered very easy by the device.

To evaluate the contextual factors for further development, we employed the QUEST, a client-centered questionnaire and outcome measure used to evaluate individual’s satisfaction with assistive technology in a structured way. It contains 12 questions, eight of them concerning characteristics of the assistive device and four questions concerning service (not appropriate for this study): *Q1 – Dimensions*, *Q2 -Weight, Q3 – Adjustments, Q4 – Safety, Q5 – Durability, Q6 – Ease of use, Q7 – Comfort, and Q8 – Effectiveness*. The individual is asked to rate the level of satisfaction with the device on a scale of 1 to 5, with 1 representing ‘not satisfied at all’ and 5 ‘very satisfied’. The total score is the mean of the individual scores (Demers et al., 2002).

As the device was not yet available as an assistive device for home use, we felt it appropriate to add the following question to the QUEST, as *Q9-Usefulness*: if possible, would you continue to use this device for your daily living? (Yes/No). The idea behind this was to abstract from eventual technical problems of the prototype, which would be a matter of product development issues, and focus on whether there is a real need for such a solution.

### Analysis

Statistical analysis was performed using MedCalc Software, Ostend, Belgium. Non-parametric summary data are calculated as median and interquartile range (IQR). We applied the paired samples Wilcoxon test to compare before and after scores of the (non-parametric) IPPA score. IPPA and QUEST scores are reported as mean and standard deviation (STD) in line with practice in literature (Wessels et al., 2002). We expected that a high IPPA score would yield a positive response on the question of *Q9-Usefulness* and applied logistic regression.

The effect size of IPPA is considered large if exceeding 0.8. For comparison, the provision of a powered wheelchair, as an essential mobility device, is reported to yield an effect size of 2.4 (Pettersson et al., 2006).

## Results

### Participants

Two hundred seventy-three clinical records were screened and 79 possibly eligible subjects came for the evaluation of the motor status and to test the device. Twenty seven of these were eligible, willing and able to participate and enrolled in the study. The device provided immediate improvement of the tenodesis grip of all participants. See further recruitment details in Thorsen et al. (2013).

Median age of the participants was 36 years (IQR: 29–41), mostly males (*n* = 24) with a median time since injury of 7 (IQR:4–16) years. The dominant hand (after lesion) was used to test the device in all cases. In many cases, also the ipsilateral hand was tested and in two cases the subjects repeated the trial off-protocol upon their request. Most common reasons for not obtaining functional grasp were: sufficiently strong tenodesis grip, lack of functional response to the stimulation, non-functional contractures or deformities.

### Experiences With Device & Electrode Setup

Setting up the devices was more difficult than foreseen in the protocol and often required technical assistance to the participants and therapists.

It was found efficient to place stimulation electrodes first to provide adequate contractions of finger/thumb flexors, effectively revealing the innervation status of these. Various anatomical textbooks were frequently consulted to predict the best electrode placement but sometimes the exact location could be different. We experienced that electrode location was very individual for each participant. Sometimes, several different electrode arrangements were tried before obtaining useful results. With one channel of stimulation available, it was found possible in most cases to balance activation of finger flexion and thumb by moving the electrodes. A recurrent problem was lack of index finger flexion (proximal interphalangeal joint) with extensive middle finger flexion resulting in inefficient key or tripod grip. Lack of adequate thumb abduction or inadequate coordination with finger flexion was also observed. As a result, eligible subjects were those, where these problems could be compensated by repositioning the electrodes.

We observed that the muscle bulk of the wrist extensors was easy to locate as it was often hypertrophic, providing a good signal and that the recording electrodes were less sensitive to precise placement. However, the best result was when they were perpendicular to the stimulation electrodes as that configuration proved to reduce stimulation responses (Thorsen et al., 2006). At times the adhesive electrodes did not provide adequate electrical contact at first application.

During this process the graphical user interface proved indispensable. Visual adjustments of parameters were preferred over the pushbuttons. The mode *–* push button on the other hand proved useful during both setup and use, allowing participants and therapists to pause the system quickly.

Overall, the initial electrode location process, for each participant, was a process of trial and error. Methods for repeating electrode positions were developed *ad hoc* using annotations, photos, skin features and/or permanent markers to allow electrode positions to be maintained for each participant during the trial.

Excessive electrode impedance was observed in the recorded signal using the GUI. It could be a strong common mode 50 Hz signal (mains interference) which saturated the signal. Or it could be a stimulation response that saturated the signal. For the system to work, the filtered signal should be close to zero during relaxation and show the volitional myoelectric signal when the wrist is extended volitionally. Skin preparations as prescribed in literature (cleaning or light abrasion) could solve the problem in most cases. Some had very dry skin. Some had hairy arms requiring shaving to improve electrode adhesion and electrical contact.

The parameter adjustment procedure was developed as follows. First, maximum stimulation was adjusted as the maximal comfortable level of stimulation, providing a firm grip. Then the gain was ramped up until a volitional wrist extension gave the participant a gradual control of the stimulation. If the stimulation did not stop when the wrist was relaxed we lowered the gain or increased the offset using the GUI. When the offset was too low the stimulation continued despite relaxation of the wrist extensors. After some iterations a proportional control of the stimulation between null output and maximum stimulation output was achieved. The offset, gain and maximum stimulation depended on what activity the user wanted to undertake and the final settings was a common decision between participant and therapist. Various everyday objects (water bottles, books, pens, soft plastic cups) were used to evaluate the usefulness of the resulting grasp. For example, to grasp a bottle the hand needs to be extended partially to place the hand around the bottle. This would require an offset to avoid premature stimulation. Then further active wrist extension could activate the stimulation gradually stimulating the fingers to flex around the bottle. Likewise a higher gain was sometimes needed to provide a firm grip before hyperextending the wrist. Parameter adjustment and sometimes also electrode placements were often reiterated before obtaining a well controlled grip. We controlled if the grip was improved by MeCFES by putting the device in pause and observing a weaker grasp force.

It happened frequently that an excessive gain caused the system to lock into a full-on stimulation state, as described in the introduction, where relaxing the wrist would not cause the stimulation to stop. Due to the limiting maximum stimulation level this was not a safety problem but an annoying issue for the user. Many participants were able to pause the system in that case, but it required an intervention of the therapist to lower the gain.

### Device Use and Outcomes

The participants exercised mostly activities which fell into the problem areas identified by the IPPA questionnaire. Over time also other activities were practiced guided by the occupational therapists.

Table 1 lists the ten most frequent types of IPPA problem areas, which the participants wanted to improve with the device. Improving writing, dressing and manipulating objects for drinking was a priority for more than half the participants, followed by cooking, cutting, heavy object manipulation, personal care, and using keys and forks. All issues were related to everyday activities in the ICF (World Health Organization [WHO], 2001) domains of general tasks, mobility, self care, domestic life, eating and personal hygiene: using an electric razor, lifting a glass and drink, opening a door, cutting and lifting heavy objects. On average the IPPA changescore was 4.6 points (STD:3.5) indicating that the device was perceived efficient for enabling ADL. This change was statistically significant (*p* < 0.001) with an effect size of 1.3. The effect was larger than 0.8 for 19 of the 27 participants (70%). Table 2 lists IPPA change scores for each participant, the *Q9-Usefulness* response and the neurological classification (ipsilateral to the hand where MeCFES was applied).

**TABLE 1:** The nine most frequent activities of daily living that the participants wished to improve.

**Activities**	**Percentage**
Writing	63%
Dressing	63%
Manipulating glasses/bottles	52%
Cooking	48%
Cutting while eating or cooking	48%
Heavy object manipulation	44%
Personal Care	41%
Using a key	37%
Using a fork	26%

**TABLE 2:** Individually prioritized problem assessments: the IPPA scores, the usefulness response, the neurological level and AIS grade of completeness for the 27 subjects.

**ID**	**IPPA Pre**	**IPPA FU**	**IPPA Score**	**Q9-Usefulness**	**Level**
N1	18	15	3	Yes	C6B
N2	10.2	6.7	3.5	Yes	C5A
N3	18	14.8	3.2	Yes	C7B
N4	15.4	10.6	4.8	No	C5A
N5	14.4	7.4	7	No	C5A
N6	13.2	7	6.2	Yes	C6A
N7	22.5	15.5	7	No	C6B
N8	23.7	18	5.7	Yes	C7A
N9	13	12	1	Yes	C6B
N10	20	13.6	6.4	Yes	C5A
N11	15.7	10.4	5.3	Yes	C6C
N12	20.8	14.2	6.8	Yes	C5B
N13	18	12.7	5.3	Yes	C6A
N14	18.2	7.2	11	No	C5A
N15	17.4	11.8	5.6	No	C5A
N16	21.4	18.4	3	No	C7B
N17	18.5	14.8	3.7	Yes	C6A
S18	18.4	8.1	10.3	Yes	C6A
S19	11.6	9	2.6	No	C7A
S20	15.3	10.4	4.9	Yes	C6A
S21	12.3	10.1	2.1	No	C6A
S22	14.3	10	4	No	C7A
S23	15	14.7	0.3	No	C7A
S24	13.6	11.3	2.3	No	C6A
S25	10.4	9.1	1.3	Yes	C6A
S26	12.4	11.4	1	No	C6A
S27	11.3	9.1	2.1	No	C7A
**Mean**	**17.0**	**11.8**	**4.6 (*p* < 0.001)**		
**SD**	**2.5**	**4.1**	**3.5**		
**Total positive**			**27**	**14**	

*The prefix for the ID indicates the spinal unit (N/S).*

Not all tasks were always facilitated by the device. For example 3 participants found the device not helpful for dining related activities, where it was perceived mostly as a hindrance. Writing (one of the highest and most often prioritized tasks) was facilitated in 3 out of the 17 subjects who had practiced this task. The others found the MeCFES assisted grasp not useful for writing.

Fourteen subjects were positive to *Q9-Usefulness*, indicating that the device would be valuable and useful for continued use at home. A logistic regression resulted in no significant correlation (*p* = 0,4) between the IPPA changescore and *Q9-Usefulness*. The table evinces some subjects (e.g. N14) having a high IPPA changescore but a negative response to Q9-Usefulness and vice versa.

Five participants had items of maximal importance which they found impossible to perform without the device. In these cases, the device changed the difficulty from impossible to easy, yielding the maximum partial change scores of 20 IPPA points.

Furthermore, some therapeutic effects were reported by some participants and therapists. Some activities had become easier also without the device. For example, one participant had learned how to load the wheelchair in his car, using MeCFES, and retained this new capability after the trial.

In some cases the natural tenodesis grip appeared stronger as participants during the trial became able to manipulate objects (e.g., those often used in OT for training ADL tasks, like water bottles, books etc.) that initially caused trouble.

The level of satisfaction with the device according to the QUEST tool, see Table 3, revealed a number of aspects that should be addressed. Firstly was the adjustments. As users, the participants were not very satisfied with the procedure of mounting and setting up the device as the procedure of adjusting the parameters gain, offset, maximum stimulation and locating the electrode positions was a cumbersome process. Secondly was the dimension of the device, by which users mostly intended the problems with tangling of the wires. At the other end, the users were fairly satisfied by the weight, effectiveness and safety. Above all, it was found easy to use.

**TABLE 3:** The items of the QUEST evaluations of the device, ordered by item score. Mean and standard deviations for each question as well as for the QUEST summary score.

**Item**	**Mean (STD)**
3. Adjustments	1.7 (0.7)
1. Dimensions	2.7 (0.9)
7. Comfort	3 (1.2)
5. Durability	3.1 (1.1)
2. Weight	3.3 (1.2)
8. Effectiveness	3.3 (1)
4. Safety	3.4 (1.1)
6. Easy to use	4.3 (1.1)
**QUEST Score**	**3.1 (0.6)**

The logistic regression between the items on the eight items of the QUEST scores and *Q9-Usefulness* isolated item *Q8-Effectiveness* as most correlated (*p* = 0.015, Wald criteria). Though possibly correlated with *Q9-Usefulness* (*p* < 0.09, Chi-squared:11), there are inconsistent answers; one participant was positive to *Q9-Usefulness* but scored *Q8-Effectiveness* as 2 and 3 participants gave a score of 4 despite being negative to *Q9-Usefulness*. When preparing the device with the electrodes already connected to the wires, some subjects were able to take and put on the electrodes by themselves and activate the device. However, they found it to be a difficult and lengthy process. Six subjects answered *NO* to *Q9-Usefulness* because they felt they already had the necessary solutions to the problems. Other 6 participants found that the system was too difficult to use due to its embodiment and method of fitting. Only one subject had a problem with comfort as finding the stimulation unpleasant.

Regarding the *Q9-Usefulness* 3 of 7 in the C5 group, 9 of 13 in the C6 group and 2 of 7 in the C7 group were positive to the utility of the device.

## Discussion

The tenodesis grip can be greatly improved by a specific application of a MeCFES device. In a prior work we have reported that it is efficient from the perspective of performance (Thorsen et al., 2013). It may be considered as a virtual tendon transfer that can strengthen the tenodesis grip. The advantage is that it is a non-invasive method that can be applied as a relatively simple assistive device.

This paper shows results of how the users perceived the device, issues in applying the system, what activities it facilitated and the usefulness. These users were people with a chronic cervical spinal cord lesion coming from two spinal cord units in northern Italy; people who had the time and possibility to participate and only those where the device immediately improved the grip.

It should be considered that the devices were prototypes built for the purpose of this research and therefore lacked some of the design features normally expected from commercial products.

Our participants tested the method as an assistive device for performing self prioritized activities in an occupational therapy setting. This trial period of totally 24 h gave the opportunity for the participants to practice various domestic activities as well as, for example, loading the wheelchair onto the car. The time allocated appeared adequate for giving the participants the experience to evaluate advantages and disadvantages as an assistive technology. Furthermore a carry-over improvement in abilities was observed.

### Candidates

People who could use this kind of neuroprosthesis as an assistive technology, enhancing the tenodesis grip, are sharing characteristics of candidates for surgical restoration of hand function (Bryden et al., 2012).

People who gained meaningful improvements and found the device useful were mainly people with a C6 lesion and to a lesser extent people with a C5 or C7 lesion. According to the ASIA classification C4 or higher should not have distal control, which does not exclude the feasibility of other MeCFES configurations. C8 lesion and below should have normal hands, wherefore augmentation of the tenodesis grip does not apply. That said, there may be special cases of incomplete injuries which could benefit.

Active wrist extension must be present. Stimulation of the finger flexors must add strength to the functional grip.

Clinical databases did not, and are not likely to contain the specific information that allows efficient selection of candidates; the status of the hand function, innervation of finger flexors and active wrist extension. People with contraindications for FES may, however, be excluded early on. As muscle stimulation devices are widely available and often used (Hayashibe, 2016), it is fairly easy for clinicians to test if the finger flexors can be stimulated to a functional tenodesis grip.

### Application of the Device

We found it difficult to establish a systematic way of placing the stimulation electrodes. The location depends on multiple factors; variations of *–* anatomy, hand posture, preservations of innervation, shape of the forearm etc. These factors are individual and optimal electrode placement had to be established for each participant at the first session. This was a time consuming process depending on each individual. For some it took up to 2 h, but in most cases about half an hour was sufficient. When stimulation is applied to the untrained muscles, fatigue may come into play and put a time limit to the process. These problems are commonly acknowledged (Koutsou et al., 2016; Sun et al., 2018). Once the settings and electrode positions were established at the first session, repeating the setup in the following sessions took around quarter of an hour. Repeatability of electrode positions and the times for setting up the system are subjects to be studied further.

One channel of stimulation was sufficient in our cases, to stimulate both long finger flexors and the thumb flexor using lateral/medial adjustments of electrode locations to adjust coordination the flexion of the forefinger vs. middle finger and the thumb flexion. Though this single channel device proved viable, multichannel systems should be investigated further. As additional channels may give better responses it should be carried in mind that it would also greatly increase the complexity of the system. It should be considered that, stimulation is activating the nerves rather than the muscle fibers themselves, that the flexor digitorum superficialis comprises four compartments with respective nerve branches, that flexors are composite muscles with variations in innervation (radial vs. median nerve) and finally the possibility of anatomical variations (Yammine and Erić, 2018). These issues combined with varying degrees of residual control of intrinsics and finger extensors makes it difficult to predict the stimulated hand function without actually stimulating at various points (Kenney et al., 2016). Moreover, racial variations are likely to exist (Al-Qattan, 2010), which calls for studies in other parts of the world.

It was generally easier to identify the recording electrodes positions than the stimulation electrodes. However, they are sensitive to stimulation artifacts and all electrodes require good electrical contact (low impedance). Recording electrodes should be perpendicular to the stimulation current path to minimize stimulation artifacts, but may be on the cost of attenuated myoelectric signal as this depends on the direction of muscle fibers.

However, we experienced that, once established for each participant, the electrode positions could be held constant for the rest of the trial. Therefore we believe that a customized mechanical solution should be developed for placing the electrodes. Preferably allowing for donning and doffing the whole system by the user in complete autonomy.

### Device Use

All participants found that the device facilitated one or more self prioritized activities. We observed that it is relatively simple, easy and directly applicable as a method of improving the hand function. It can address and solve central priorities for many people with spinal cord injury; with a positive impact on facilitating activities in their daily lives.

The IPPA effect size is large and can be compared to what, for example, could be expected from providing a mobility device (Wessels et al., 2004). However, not all participants found that the device in its present form would be useful for home use, even if it was available. The IPPA change score, despite the fact that it reflects the users priorities, did not correlate well with the perceived usefulness. Each participant had an individual explanation for this. Most typical was the unwieldiness of the device, problems with wires and electrode placement.

Many individual problems were evinced by the IPPA and could be generalized into the ICF domains of fine hand use, arm-hand, basic ADL and extended ADL. Some activities as for example dressing, involves multiple types of manipulation and activities like writing, calls for complex grips i.e. the tripod grip. Such fine motor functions are difficult to control via simple surface stimulation, which may explain why this was one of the priorities that the device presently did not facilitate well. Future development should specifically address this grip, but taking into consideration that people may use alternative grasps instead of the tripod grip for writing.

The complexity of successful development of assistive technology is evident from the inconsistency between IPPA priorities and perceived usefulness.

Most participants were satisfied with the safety, ease of use and effectiveness of the device. That was expected since one of the strengths of the method is that it should be very intuitively easy to use. There were issues with adjustments and dimensions. The latter can be attributed to the wire tangling problems. Safety problems were explained by problems with electrodes peeling off and thus rather reflected problems related to reliability of the device. No health hazard or risk problems were reported. Effectiveness appears to be correlated to the likelihood of perceiving the device useful for home use. However, the sample size is not providing the power to confirm this.

It may also be considered that users may want or need bilateral devices. Therefore possible interactions of multiple devices should be assessed in further studies.

### Other Uses

We envisage other possible applications of this method. As we observed a possible therapeutic effect it may become useful as a training device during occupational therapy. Whether new abilities that the participants acquired are persistent as well as if the cause is increasing muscle strength (Patil et al., 2015), neural recovery/plasticity or a result of learning new ways of manipulate things (Curt et al., 2008), remains to be investigated further but would involve large randomized controlled clinical studies. However, even small improvements in hand function can lead to significant increase in functional independence in daily living for people with tetraplegia (Patil et al., 2015).

Yet, another application could be as a simulator for reconstructive surgery in order to let the candidate make a more informed decision on what functional gains they may obtain (Dunn et al., 2012). Finally we may speculate that the availability of such a device in the future could influence the paradigm of conservative treatment early on in rehabilitation, as it may open up for preparing the hands for prospective use of neuroprosthetic assistive technologies.

### Prospective Development

Ideally the user should not have to bother with cables and manually placing electrodes with each use. Implanting such systems are being investigated with additional advantages such as better muscle selectivity and reliability (Memberg et al., 2014). However, implants are still in their infancy, challenged by cost, invasiveness, risk of surgery and are still being researched (Tigra et al., 2019). FreeHand system (Neurocontrol Corp., United States) was a good example of this kind of device which has been commercially available for a period, but it was eventually dismissed.

To render MeCFES assisted tenodesis grip clinically useful, someone will eventually have to produce and market the device. Besides resolving the technical issues of wearability, design and facilitating setup, technology transfer of research devices, faces several obstacles (Peckham et al., 1996). Marketing and finding suitable candidates is a complex issue; clinical records lack information needed to target users, clinicians and clients will need to be taught about the possibilities and limitations, devices must be available for rehabilitators and patients to try. Specific knowledge must be disseminated as it’s not similar to other assistive devices. Clinicians must be trained to know how the device can be applied, how to identify electrode locations and in the skills to quickly set-up the device for testing eligibility of their client. Therefore a manufacturer may face investments incurring a final price which results too unfavorable for both provider and user.

That leaves a void where there are thousands of people who could become enabled in their prioritized activities of daily living using this non-invasive technique, but there is no way for them to acquire the necessary equipment nor knowing the existence of the possibility.

To overcome these issues we are currently in the process of investigating the options of using co-design and open source hardware and software as a means to disseminate the method in collaboration with people with spinal cord injury, clinicians and technicians. Currently the material cost of such a prototype is below 200€^1^. Such participative design may ensure that critical issues outlined by this paper, device design, wearability, user interfacing and control will be addressed (Thorsen et al., 2019).

## Conclusion

We have demonstrated that a device for myoelectrically controlled functional electrical stimulation can be an efficient assistive device for activities of daily living. A research prototype was used to provide the effect of a virtual tendon transfer to improve the tenodesis grip and facilitate self-selected activities. Users found it safe, effective, intuitive and easy to use. Fourteen of the 27 participants in the trial found the prototype so useful that they would like to continue using it after the trial. A number of issues related to the embodiment of the prototype were identified; wearability and ease of fitting should be improved.

Further work should be done to solve these issues because such a neuroprosthesis could enable autonomy in activities and participation in daily life in a meaningful way for many people with tetraplegia.

## Data Availability Statement

The raw data supporting the conclusions of this article will be made available by the authors, without undue reservation.

## Ethics Statement

The studies involving human participants were reviewed and approved by the following local medical ethics committees: Fondazione don Carlo Gnocchi Onlus (16 October 2007) Azienda Ospedaliera Ospedale, Niguarda Ca’ Granda di Milano (13 December 2007), Ente Ospedaliero di Bormio e Sondalo “E. Morelli” (30 November 2007). The patients/participants provided their written informed consent to participate in this study.

## Author Contributions

RT contributed to the manuscript, research idea, production of materials, and project and data management. DD contributed to the manuscript revisions, clinical supervision, and data collection. EB contributed to the critical appraisal and revision of the manuscript. MF contributed to the manuscript revisions, project planning, and funding. All authors contributed to the manuscript revision, read and approved the submitted version.

## Conflict of Interest

The authors declare that the research was conducted in the absence of any commercial or financial relationships that could be construed as a potential conflict of interest.

## Footnotes

1 https://thorsenrune.github.io/MeCFES

## References

[B1] Al-Qattan M. M. (2010). Variations in the course of the thenar motor branch of the median nerve and their relationship to the hypertrophic muscle overlying the transverse carpal ligament. *J. Hand Surg.* 35 1820–1824. 10.1016/j.jhsa.2010.08.011 20934817

[B2] American Spinal Injury Association, (1992). *International Medical Society of Paraplegia. International Standards for Neurologic And Functional Classification Of Spinal Cord Injury.* Chicago, IL: ASIA/IMSOP.

[B3] Anderson K. D. (2004). Targeting recovery: priorities of the spinal cord-injured population. *J. Neurotrauma* 21 1371–1383. 10.1089/neu.2004.21.1371 15672628

[B4] Benton L. A. Baker L. L. Bowman B. R. Waters R. L. (1980). *Functional Electrical Stimulation—A Practical Clinical Guide.* Downey, CA: Rancho Los Amigos National Rehabilitation Center.

[B5] Bromley I. (2006). *Tetraplegia and Paraplegia: A Guide For Physiotherapists.* Amsterdam: Elsevier.

[B6] Bryden A. Peljovich A. Hoyen H. Nemunaitis G. Kilgore K. Keith M. (2012). Surgical restoration of arm and hand function in people with tetraplegia. *Top. Spin. Cord Injury Rehabil.* 18 43–49. 10.1310/sci1801-43 23459698 PMC3584747

[B7] Curt A. Van Hedel H. J. Klaus D. Dietz V. EM-Science Study Group, (2008). Recovery from a spinal cord injury: significance of compensation, neural plasticity, and repair. *J. Neurotrauma* 25 677–685. 10.1089/neu.2007.0468 18578636

[B8] de Witte L. Steel E. Gupta S. Ramos V. D. Roentgen U. (2018). Assistive technology provision: towards an international framework for assuring availability and accessibility of affordable high-quality assistive technology. *Disabil. Rehabil. Assist. Technol.* 13 467–472. 10.1080/17483107.2018.1470264 29741965

[B9] Demers L. Weiss-Lambrou R. Ska B. (2002). The quebec user evaluation of satisfaction with assistive technology (QUEST 2.0): an overview and recent progress. *Technol. Disabil.* 14 101–105. 10.3233/tad-2002-14304

[B10] Dunn J. A. Hay-Smith E. J. C. Whitehead L. C. Keeling S. (2012). Issues influencing the decision to have upper limb surgery for people with tetraplegia. *Spinal Cord* 50:844. 10.1038/sc.2012.58 22584282

[B11] Hayashibe M. (2016). Evoked electromyographically controlled electrical stimulation. *Front. Neurosci.* 10:335 10.3389/fnins.2016.00335 PMC4943954 27471448

[B12] Hermens H. J. Freriks B. Disselhorst-Klug C. Rau G. (2000). Development of recommendations for SEMG sensors and sensor placement procedures. *J. Electromyogr. Kinesiol.* 10 361–374. 10.1016/s1050-6411(00)00027-4 11018445

[B13] Jonsdottir J. Thorsen R. Aprile I. Galeri S. Spannocchi G. Beghi E. (2017). Arm rehabilitation in post stroke subjects: a randomized controlled trial on the efficacy of myoelectrically driven FES applied in a task-oriented approach. *PLoS One* 12:e0188642. 10.1371/journal.pone.0188642 29200424 PMC5714329

[B14] Kenney L. P. Heller B. W. Barker A. T. Reeves M. L. Healey J. Good T. R. (2016). A review of the design and clinical evaluation of the ShefStim array-based functional electrical stimulation system. *Med. Eng. Phys.* 38 1159–1165. 10.1016/j.medengphy.2016.08.005 27639656

[B15] Koutsou A. D. Moreno J. C. del Ama A. J. Rocon E. Pons J. L. (2016). Advances in selective activation of muscles for non-invasive motor neuroprostheses. *J. Neuroeng. Rehabil.* 13:56. 10.1186/s12984-016-0165-2 PMC4907085 27296478

[B16] Memberg W. D. Polasek K. H. Hart R. L. Bryden A. M. Kilgore K. L. Nemunaitis G. A. (2014). Implanted neuroprosthesis for restoring arm and hand function in people with high level tetraplegia. *Arch. Phys. Med. Rehabil.* 95 1201–1211. 24561055 10.1016/j.apmr.2014.01.028 PMC4470503

[B17] Mulcahey M. J. Hutchinson D. Kozin S. (2007). Assessment of upper limb in tetraplegia: Considerations in evaluation and outcomes research. *J. Rehabil. Res. Dev.* 44 91–102. 17551863 10.1682/jrrd.2005.10.0167

[B18] Patil S. Raza W. A. Jamil F. Caley R. O’Connor R. J. (2015). Functional electrical stimulation for the upper limb in tetraplegic spinal cord injury: a systematic review. *J. Med. Eng. Technol.* 39 419–423. 10.3109/03091902.2015.1088095 26414202

[B19] Peckham P. H. Thrope G. Woloszko J. Habasevich R. Scherer M. Kantor C. (1996). Technology transfer of neuroprosthetic devices. *J. Rehabil. Res. Dev.* 33 173–183. 8724172

[B20] Pettersson I. Törnquist K. Ahlström G. (2006). The effect of an outdoor powered wheelchair on activity and participation in users with stroke. *Disabil. Rehabil. Assist. Technol.* 1 235–243. 10.1080/17483100600757841 19260171

[B21] Salatino C. Pigini L. Andrich R. (2018). “How to measure the impact of assistive technology solutions on the person’s quality of life?,” in *Proceedings of the 4th EAI International Conference on Smart Objects and Technologies for Social Good*, (New York, NY: ACM), 238–242.

[B22] Snoek G. J. IJzerman M. J. Hermens H. J. Maxwell D. Biering-Sorensen F. (2004). Survey of the needs of patients with spinal cord injury: impact and priority for improvement in hand function in tetraplegics. *Spinal Cord* 42 526–532. 10.1038/sj.sc.3101638 15224087

[B23] Sun M. Smith C. Howard D. Kenney L. Luckie H. Waring K. (2018). FES-UPP: a flexible functional electrical stimulation system to support upper limb functional activity practice. *Front. Neurosci.* 12:449 10.3389/fnins.2018.00449 PMC6041417 30026683

[B24] Thorsen R. (1999). An artefact suppressing fast-recovery myoelectric amplifier. *IEEE Trans. Biomed. Eng.* 46 764–766. 10.1109/10.764955 10356884

[B25] Thorsen R. Binda L. Chiaramonte S. Dalla Costa D. Redaelli T. Occhi E. (2014). Correlation among lesion level, muscle strength and hand function in cervical spinal cord injury. *Eur. J. Phys. Rehabil. Med.* 50 31–38. 23820875

[B26] Thorsen R. Bortot F. Caracciolo A. (2019). From patient to maker-a case study of co-designing an assistive device using 3D printing. *Assist. Technol.* 14, 1–7. 10.1080/10400435.2019.1634660 31237803

[B27] Thorsen R. Carpinella I. Ferrarin M. (2005). Can the F-response be volitionally repressed during functional electrical stimulation? *Neuromodul. Technol. Neural Interf.* 8 141–147. 10.1111/j.1525-1403.2005.00230.x 22151443

[B28] Thorsen R. Dalla Costa D. Chiaramonte S. Binda L. Beghi E. Redaelli T. (2013). A noninvasive neuroprosthesis augments hand grasp force in individuals with cervical spinal cord injury: the functional and therapeutic effects. *Sci. World J.* 2013:7. 10.1155/2013/836959 PMC3893005 24489513

[B29] Thorsen R. Ferrarin M. (2009). Battery powered neuromuscular stimulator circuit for use during simultaneous recording of myoelectric signals. *Med. Eng. Phys.* 31 1032–1037. 10.1016/j.medengphy.2009.06.006 19620017

[B30] Thorsen R. Spadone R. Ferrarin M. (2001). A pilot study of myoelectrically controlled FES of upper extremity. *IEEE Trans. Neural. Syst. Rehabil. Eng.* 9 161–168. 10.1109/7333.928576 11474969

[B31] Thorsen R. A. (1997). *Restoration of Hand Function In Tetraplegics Using Myoelectrically Controlled Functional Electrical| Stimulation Of The Controlling Muscle.* Doctoral dissertation, Technical University of Denmark, Denmark.

[B32] Thorsen R. A. Occhi E. Boccardi S. Ferrarin M. (2006). Functional electrical stimulation reinforced tenodesis effect controlled by myoelectric activity from wrist extensors. *J. Rehabil. Res. Dev.* 43:247. 10.1682/jrrd.2005.04.0068 16847791

[B33] Tigra W. Azevedo C. Teissier J. Gelis A. Coulet B. Divoux J. L. (2019). Implanted nerve electrical stimulation allows to selectively restore hand and forearm movements in patients with a complete Tetraplegia. *bioRxiv* [Preprint], 10.1101/534362

[B34] Wessels R. D. De Witte L. P. Jedeloo S. van den Heuvel W. P. van den Heuvel W. J. (2004). Effectiveness of provision of outdoor mobility services and devices in The Netherlands. *Clin. Rehabil.* 18, 371–378. 15180120 10.1191/0269215504cr755oa

[B35] Wessels R. Persson J. Lorentsen O. Andrich R. Ferrario M. Oortwijn W. (2002). IPPA: individually prioritised problem assessment. *Technol. Disabil.* 14 141–145. 10.3233/tad-2002-14310

[B36] Winslow C. Rozovsky J. (2003). Effect of spinal cord injury on the respiratory system. *Am. J. Phys. Med. Rehabil.* 82, 803–814. 14508412 10.1097/01.PHM.0000078184.08835.01

[B37] World Health Organization [WHO], (2001). *International Classification Of Functioning, Disability And Health: ICF.* Geneva: World Health Organization.

[B38] Yammine K. Erić M. (2018). Linburg–Comstock variation and syndrome. A meta-analysis. *Surg. Radiol. Anat.* 40 289–296. 10.1007/s00276-017-1957-1 29218383

[B39] Yarkony G. M. Roth E. J. Heinemann A. W. Lovell L. Wu Y. C. (1988). Functional skills after spinal cord injury rehabilitation: three-year longitudinal follow-up. *Archiv. Phys. Med. Rehabil.* 69 111–114. 3341888

